# Would I lie to you? Party affiliation is more important than Brexit in processing political misinformation

**DOI:** 10.1098/rsos.220508

**Published:** 2023-02-01

**Authors:** Toby Prike, Robert Reason, Ullrich K. H. Ecker, Briony Swire-Thompson, Stephan Lewandowsky

**Affiliations:** ^1^ School of Psychological Science, University of Western Australia, Perth, Australia; ^2^ Public Policy Institute, University of Western Australia, Perth, Australia; ^3^ School of Psychological Science, University of Bristol, Bristol, UK; ^4^ Network Science Institute, Northeastern University, Boston, MA, USA; ^5^ Institute of Quantitative Social Science, Harvard University, Cambridge, MA, USA; ^6^ Department of Psychology, University of Potsdam, Potsdam, Germany

**Keywords:** misinformation, fact-checking, political attitudes, belief change, voting behaviour, Brexit

## Abstract

In recent years, the UK has become divided along two key dimensions: party affiliation and Brexit position. We explored how division along these two dimensions interacts with the correction of political misinformation. Participants saw accurate and inaccurate statements (either balanced or mostly inaccurate) from two politicians from opposing parties but the same Brexit position (Experiment 1), or the same party but opposing Brexit positions (Experiment 2). Replicating previous work, fact-checking statements led participants to update their beliefs, increasing belief after fact affirmations and decreasing belief for corrected misinformation, even for politically aligned material. After receiving fact-checks participants had reduced voting intentions and more negative feelings towards party-aligned politicians (likely due to low baseline support for opposing party politicians). For Brexit alignment, the opposite was found: participants reduced their voting intentions and feelings for opposing (but not aligned) politicians following the fact-checks. These changes occurred regardless of the proportion of inaccurate statements, potentially indicating participants expect politicians to be accurate more than half the time. Finally, although we found division based on both party and Brexit alignment, effects were much stronger for party alignment, highlighting that even though new divisions have emerged in UK politics, the old divides remain dominant.

## 1. Introduction

There is considerable evidence that misinformation can have wide ranging negative consequences, for both individuals and the broader public [1,2]. For instance, within democratic political systems, the public use their voting power to elect their desired political representatives and leaders. Many normative accounts posit that, for a democracy to function, it is crucial for voters to be well informed and make informed decisions [3,4]. However, political misinformation undermines this process by introducing misleading or inaccurate information that may influence voters. Accordingly, recent years have seen a huge growth in research into misinformation as well as the effectiveness of fact-checking [1,5].

Previous research has examined the effects of political misinformation and fact-checking, including differences in how beliefs and political attitudes change depending on whether the misinformation is presented by a politician who supports or opposes a person's viewpoint [6–8]. However, this research has focused primarily on political divisions along party lines (e.g. Democrat versus Republican) rather than other ideological divisions. To investigate issue-based political divisions, we focused on the UK, which hosts perhaps the most notable issue-based political divide to emerge in recent years, the divide over Brexit [9]. Specifically, we examined the extent to which statements from politicians that were subsequently fact-checked impacted belief and political attitudes towards politicians, and the extent to which this impact varied depending on whether participants were aligned with (or opposed to) the politicians on the dimensions of political party (Labour Party/Conservative Party) and Brexit position (Leave/Remain).

### 1.1. Political misinformation and correction

A well-established finding that raises concerns about the ability of fact-checks and corrections to undo the impact of misinformation is the continued influence effect—a tendency for people to continue to be influenced by misinformation after it has been corrected (for a review, see [10]). In standard tasks used to investigate the continued influence effect, participants are shown various claims that contain inaccuracies or misinformation and rate their belief in those claims. Participants are subsequently presented with a correction, informing them that the initial misinformation was inaccurate, and they then rerate their belief in the claims. These corrections are generally effective, leading to lower belief in the misinformation than when corrections are not presented. However, corrections do not completely undo the impact of being exposed to misinformation: following a correction, misinformation belief generally persists at a higher level than if the misinformation was never presented. Additionally, even if misinformation beliefs are somewhat effectively reduced following a correction, there is often greater continued reliance on misinformation when beliefs are indirectly measured, such as via inferential reasoning questions [11,12].

When it comes to political misinformation, an additional concern arises from the fact that people often have strong political attachments, and thus motivated reasoning may make them resistant to belief updating when misinformation from politicians they support is corrected [13–15]. Fortunately, however, research into political misinformation has generally found that corrections and fact-checks are effective at decreasing belief in misinformation and increasing belief in accurate statements, at least when these beliefs are measured directly [6–8,16–18]. Swire *et al*. [7] and Nyhan *et al*. [17] both demonstrated that supporters and non-supporters of Donald Trump adjusted their level of belief in Trump statements in response to fact-checks. In a follow-up study, Swire-Thompson *et al*. [8] found that this replicated across the political aisle, with supporters and non-supporters of Donald Trump and Bernie Sanders both adjusting their belief in statements from these politicians in response to fact-checks. Aird *et al*. [6] found the same pattern in an Australian context. In sum, there is evidence to suggest that fact-checking is effective at reducing belief regardless of political affiliation, cultural context or support for the source.

However, the evidence is less clear when it comes to how fact-checking misinformation impacts political attitudes and voting intentions. Although trust in politicians is low, the general public also highly value honesty in their politicians [19,20]. The high value placed on honesty suggests that voters should have more negative views of politicians who make inaccurate statements, yet this was not borne out in the initial studies into the impact of fact-checking. Swire *et al*. [7] and Nyhan *et al*. [17] both found that presenting inaccurate statements from Donald Trump that were subsequently fact-checked had no impact on participants' feelings towards Trump or their voting intentions, even though participants understood and accepted the corrections. The follow-up studies by Aird *et al*. [6] and Swire-Thompson *et al*. [8] varied the proportion of true and false statements, in an Australian and US population, respectively. Swire-Thompson *et al*. [8] found that participants who were shown mostly inaccurate statements reduced their support even of a favoured politician, but only very slightly $\left(\eta_{p}^{2}=0.01\right)$. Aird *et al*. [6] found the same pattern in Australia, but with an effect size six times greater than that in the US study, which suggests that Australians place greater importance on the honesty of their politicians and are more willing to adjust their attitudes towards politicians. The differences in how USA and Australian participants respond to corrections of political misinformation highlight that culture and political climate may play an important role, emphasizing the need to examine political misinformation in additional cultural contexts.

### 1.2. The present study

The UK provides an interesting context in which to study the interactions between political support and political misinformation because in recent years it has seen political divisions along a new dimension, *viz*. attitudes towards Brexit [9]. In 2013, in an effort to fend off challenges from the UK Independence Party and placate the Eurosceptic faction of the Conservative Party, former Prime Minister David Cameron added a commitment to hold a referendum on the UK's membership of the European Union to the Conservative Party election manifesto [21]. In the lead up to the Brexit referendum, the leadership of both major parties campaigned for the UK to remain in the EU, although many Conservative Party MPs and a few Labour Party MPs supported the campaign for the UK to leave the EU [22]. Then, following the 2016 referendum result in favour of leave, both the centre-left Labour and the centre-right Conservative party committed to taking the UK out of the EU during the 2017 election campaign. Additionally, although Pew Research surveys showed differences in Brexit attitudes between Labour (majority support remain) and Conservative party supporters (majority support leave), there are still sizeable minorities of both parties' supporters who hold the opposing Brexit stance [23,24]. These patterns were also reflected within the members of parliament representing the two major parties, meaning for several years after the referendum there were some remain-supporting Conservative MPs and some leave-supporting Labour MPs. Now that the UK has left the EU, most MPs have accepted the leave result and want to move on to other issues [25].

In the current study, we examined political misinformation in the UK. Specifically, we examined how participants updated their beliefs in accurate (fact) and inaccurate (myth) statements from politicians when presented with fact-checks. All statements were authentic (i.e. had actually been made by politicians). Additionally, we examined whether presenting the statements and fact-checks impacted participants’ attitudes towards politicians. In line with Aird *et al*. [6] and Swire-Thompson *et al*. [8], we also examined whether belief updating and changes in attitudes varied depending on support for the politician and if the proportion of inaccurate to accurate statements (equal or disproportionately inaccurate) mattered. Because politics within the UK, in addition to traditional divisions along party lines, has also become divided according to Brexit position, we operationalized those two dimensions by including four politicians as statement sources who occupied all possible combinations of party and Brexit identity: Jess Phillips (a Labour remainer), Kate Hoey (a Labour leaver), Nick Boles (a Conservative remainer) and Chris Grayling (a Conservative leaver). To better investigate the relative impact of divisions along party and Brexit lines, greater emphasis was placed on party affiliation in Experiment 1, whereas in Experiment 2 greater emphasis was placed on Brexit position. One important caveat is that although the politicians' biographies mentioned their party affiliation in both experiments, their Brexit positions were only mentioned in Experiment 2. Party affiliation was mentioned across both experiments to enhance realism. Specifically, when a politician is mentioned or quoted in the media their party affiliation is usually also noted, and party affiliations are always listed next to a politician's name on a ballot. Additionally, these politicians were selected because they were well known, active in the Brexit debate, and had similar levels of Internet search activity (see the Materials and Procedure sections for more details).

### 1.3. Research questions

The key research questions for this study were:

1. Whether UK participants would respond to fact-checks in the same way as those in the USA and Australia, that is, by increasing their belief in accurate statements and decreasing their belief in inaccurate statements, regardless of whether the source is politically aligned.
2. How participants’ political attitudes (i.e. voting intentions, feelings of warmth and perceived veracity of politicians' claims) would change in response to the presentation of the statements and fact-checks—in other words, whether UK voters’ regard for politician honesty was more in line with the USA or Australia.
3. Whether presenting mostly inaccurate statements would lead to lower statement belief and more negative political attitudes.
4. Whether party affiliation or Brexit position would have a stronger influence on participants' beliefs and attitudes, including how these beliefs and attitudes change in response to the presentation of the statements and fact-checks.

## 2. Method

Although we collected data for two separate but highly similar studies, to facilitate presentation we describe them as a single study. Both experiments had within-between designs. The key factors of interest were pre/post (pre fact-check, post fact-check), myth proportion (balanced, mostly myths), party congruence (congruent, incongruent) and Brexit-position congruence (congruent, incongruent).

### 2.1. Participants

Participants were residents of the UK recruited via Prolific and were reimbursed £1.40 for completing the 15-min study. The study was administered using Qualtrics survey software. Based on the sample size used in Aird *et al*. [6], we recruited 394 participants in Experiment 1 and 395 participants in Experiment 2. Following an *a priori* analysis plan, participants were excluded if they incorrectly answered either of two basic political check questions (see Materials below; Experiment 1: *n* = 51; Experiment 2: *n* = 56)^1^ or self-reported that they had not paid attention (Experiment 1: *n* = 3; Experiment 2: *n* = 2). Because we were interested in the effects of political divisions, we also removed participants who were not supporters of Labour or the Conservative party and/or did not have a position on Brexit (Experiment 1: *n* = 92; Experiment 2: *n* = 115). This left us with final samples of *N* = 248 in Experiment 1 (166 Labour supporters; 82 Conservative supporters; 173 remainers; 75 leavers) and *N* = 222 in Experiment 2 (161 Labour supporters; 61 Conservative supporters; 169 remainers; 53 leavers). The Experiment 1 sample had the following demographic characteristics: *M*_age_ = 36.02; age range 18–72; 99 males; 147 females; 3 individuals who selected other. Concerning education, 4 did not graduate from high school; 26 were high school graduates; 68 with A or AS level; 103 held a university degree; and 47 had a postgraduate degree. The Experiment 2 sample had the following demographic characteristics: *M*_age_ = 35.15; age range 18–76; 68 males; 153 females; 1 individual who selected other. Concerning education, 1 did not graduate from high school; 26 were high school graduates; 59 with A or AS level; 99 held a university degree; and 37 had a postgraduate degree. Data were collected in March and April 2020.

### 2.2. Materials

True and false statements from four British MPs were used. To this end, politicians with similar levels of news-related search activity on Google were selected (see electronic supplementary material, figure S1), with two from the Labour Party: Jess Phillips and Kate Hoey, and two from the Conservative Party: Chris Grayling and Nick Boles. Eight statements were collected for each politician from interviews, parliamentary transcripts, social media and newspaper articles. Four statements were true and four were false (for examples, table 1; all statements are provided at https://osf.io/tnzsa/); statements were verified through use of fact-checking websites and governmental reports. Short biographies were also written for each politician. These biographies noted their party affiliation and in Experiment 1 emphasized their domestic political record, whereas in Experiment 2 their Brexit stance was emphasized (see https://osf.io/tnzsa/). Participants completed a questionnaire containing the statements (all eight statements in the balanced condition, four false and one true in the mostly myths condition), with statements blocked by politician. The order of the politicians was counter-balanced and the presentation of statements within each block was randomized. Each statement was rated on a scale from ‘Definitely False’ (0) to ‘Definitely True’ (5).

**Table 1.** Examples of misinformation statements and associated corrections. Note. For all statements see https://osf.io/tnzsa/.

politician	misinformation	correction
Nick Boles (Conservative Politician)	Nick Boles said that ‘Reporters without Borders… ranks Israel higher than the United States as a place for press freedom’.	This is False Israel has never been ranked higher than the USA, the lowest US finish in the last 10 years has been 49th, while Israel's highest placement has been 87th. You previously rated this question a X out of 5 (0 = definitely false, 5 = definitely true).
Kate Hoey (Labour Politician)	In 2013, Kate Hoey said that ‘the London skyline has changed hugely over the last few years, the number of helicopters flying have increased a great deal’	This is False Civil Aviation Authority Statistics in December 2007 showed an average of 58 flights per day in London, with a peak of 143 in June 2008. By 2013, this average was down to 36.5, with monthly figures showing a steady decrease. You previously rated this question a X out of 5 (0 = definitely false, 5 = definitely true).

Three measures of political attitude were also collected: voting intentions for each politician, feelings towards each politician, and proportion of inaccurate claims made by each politician. To assess voting intentions, participants indicated how likely they would be to vote for each politician using a scale ranging from ‘Not at all Likely’ (0) to ‘Very Likely’ (5). Following Swire-Thompson *et al*. [7], feelings towards each politician were assessed using a ‘feeling thermometer’, on a scale ranging from 0 to 100. Participants were informed that ratings between 0 and 49 signified a lack of warmth, whereas ratings between 51 and 100 implied a feeling of warmth; ratings of 50 suggested indifference. Finally, judgements of the proportion of inaccurate claims politicians make were assessed by asking participants what percentage of day-to-day claims made by each politician they estimate to be inaccurate. Political knowledge was also checked using two multiple-choice questions: ‘who is the current Prime Minister?’ and ‘what was the result of the 2019 General Election?’.

### 2.3. Procedure

**Figure 1.**
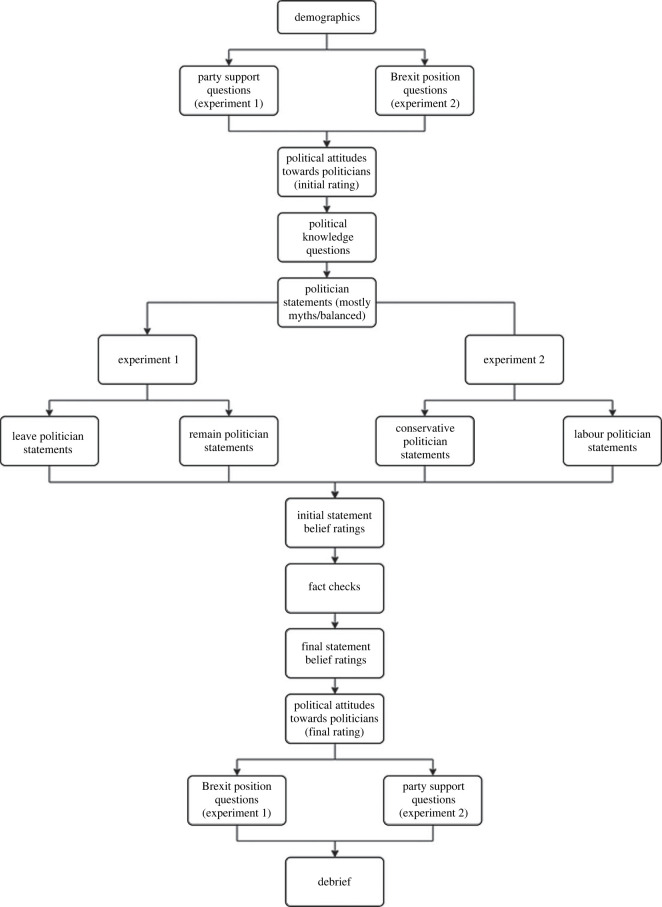
Flowchart outlining the study procedure.

The procedure is outlined in figure 1. Participants began by reviewing a consent form approved by the University of Bristol. Basic demographic information concerning age, gender, education level, interest in politics and self-reported knowledge of politics was then collected. In Experiment 1, participants were then asked which party they support using a multiple-choice question with the options Conservative, Labour Party, Liberal Democrats and other (if other was selected participants could type in the party they support). In Experiment 2, participants were instead asked about their Brexit stance using a multiple-choice question with the options leave, remain and undecided. Participants then indicated their political attitudes towards the four politicians of interest: Kate Hoey, Jess Phillips, Chris Grayling, Nick Boles and the party leaders at the time, Boris Johnson and Jeremy Corbyn.

Political knowledge was then assessed using the two political check questions. Following this, participants were assigned to one of two conditions. In Experiment 1, these conditions each presented statements from one Conservative and one Labour politician of the same Brexit position (i.e. Remain Condition: Boles and Phillips; Leave Condition: Grayling and Hoey). In Experiment 2, these conditions each presented statements from two members of the same political party who differed in their Brexit stance (i.e. Conservative Condition: Boles and Grayling; Labour Condition: Phillips and Hoey). Participants were then presented with either five (four myths but only one fact) or eight (four myths and four facts) statements for each politician. Following the initial presentation of each statement, participants rated their belief in the statement. A fact-check affirming or correcting each statement was then presented, accompanied by a reminder of the participant's initial belief rating. Each statement was then presented a final time for a second belief rating. Participants then completed a second round of voting intentions, feelings of warmth and proportion of inaccurate claims measures before being asked the political affiliation questions they had not yet completed (Brexit position in Experiment 1, party affiliation in Experiment 2). Finally, participants self-reported their level of attention during the survey and were fully debriefed.

## 3. Results

We were interested in the effects of political divisions along the lines of political party and Brexit position. Although the emphasis differed between the two experiments, with Experiment 1 placing greater emphasis on party affiliation and Experiment 2 greater emphasis on Brexit position, it was still possible to analyse both types of political division within each experiment. To simplify the design, party support and politician were recoded as party congruence (i.e. does the participant support the party that politician is from) and Brexit position and politician were recoded as Brexit congruence (i.e. does the participant hold the same Brexit position as the politician). We then conducted two separate mixed ANOVAs for each experiment. The first analysis examined the impact of whether participants and politicians were of the same or opposing political parties; the mixed ANOVA included pre/post (pre fact-check, post fact-check) as a within-subjects factor, myth proportion (balanced, mostly myths) as a between-subjects factor, and party congruence (congruent, incongruent; within-subjects in Experiment 1, between-subjects in Experiment 2). The second analysis examined whether participants and politicians shared the same position on Brexit; the mixed ANOVA included pre/post (pre fact-check, post fact-check) as a within-subjects factor, myth proportion (balanced, mostly myths) as a between-subjects factor and Brexit congruence (congruent, incongruent; between-subjects in Experiment 1, within-subjects in Experiment 2). Because the Brexit congruence analyses were conducted on the same data as the party congruence analyse, when reporting the Brexit congruence analyses, we report only the main effect of Brexit congruence as well as any interactions it is involved in without rereporting the results for pre/post and/or myth proportion, to avoid unnecessary repetition.

### 3.1. Statement beliefs and inaccurate claims

**Figure 2.**
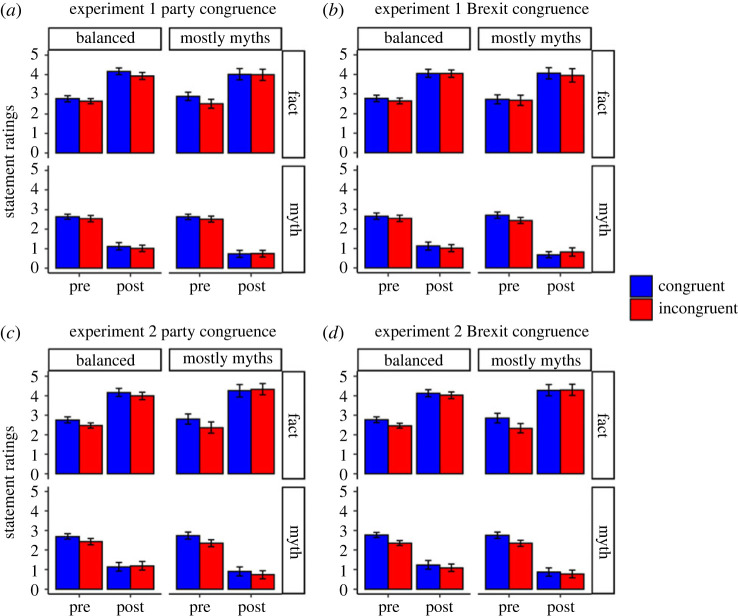
Statement-belief ratings for experiments 1 and 2. *Note.* Error bars represent 95% confidence intervals. Results show that overall, fact-checks worked: belief in facts increased from Pre to Post, and belief in myths decreased. There is also an effect of party congruence, with greater belief when participants supported the party of the politicians making the statements. A similar effect of Brexit-position congruence was significant only in Experiment 2. See text for additional details.

To address our research questions about the impact of fact-checks on beliefs, we first analysed how participants updated their belief in the fact and myth statements in response to the fact-checks, as well as if this differed depending on political congruence and the proportion of fact and myth statements. Statement-belief ratings for both experiments are presented in figure 2 (also see electronic supplementary material, tables S2–S5 for descriptive statistics). Statement-belief ratings for fact and myth statements were analysed separately.

#### 3.1.1. Fact belief ratings

In Experiment 1, for belief in the factual statements, there were significant main effects of pre/post, *F*_1, 246_ = 367.50, *p* < 0.001, $\eta_{p}^{2}=0.60$, 95% CI [0.53, 0.66], with higher belief post fact-check, and of party congruence, *F*_1, 246_ = 5.55, *p* = 0.019, $\eta_{p}^{2}=0.02$, 95% CI [0.00, 0.07], with higher belief when politicians and participants were aligned with the same political party. There was no significant main effect of myth proportion, nor any interactions. There was also no significant main effect of Brexit congruence and it was not involved in any significant interactions.

The pre/post and party congruence main effects in Experiment 2 were replicated (*p* < 0.001 and *p* = 0.013, respectively, see electronic supplementary material for full analyses). Unlike in Experiment 1, there was a significant main effect of Brexit congruence, *F*_1, 220_ = 8.72, *p* = 0.003, $\eta_{p}^{2}=0.04$, 95% CI [0.00, 0.10], with higher belief in statements from politicians with the same Brexit position. However, this was qualified by a significant pre/post by Brexit congruence interaction, *F*_1, 220_ = 7.16, *p* = 0.008, $\eta_{p}^{2}=0.03$, 95% CI [0.00, 0.09]. Follow-up paired *t*-tests revealed that participants significantly increased their belief in factual statements following the fact-checks for both Brexit-congruent, *t*_221_ = 12.90, *p* < 0.001, *d* = 0.87, 95% CI [0.69, 1.07], and Brexit-incongruent politicians, *t*_221_ = 16.50, *p* < 0.001, *d* = 1.11, 95% CI [0.92, 1.36]; however, this effect was larger for Brexit-incongruent politicians due to the lower pre-affirmation baseline. There was no main effect of myth proportion, and it was not involved in any interactions.

#### 3.1.2. Myth belief ratings

In Experiment 1, for belief in the myth statements, there were significant main effects of pre/post, *F*_1, 246_ = 891.82, *p* < 0.001, $\eta_{p}^{2}=0.78$, 95% CI [0.74, 0.82], with higher belief prior to the fact-checks, and myth proportion, *F*_1, 246_ = 5.93, *p* = 0.016, $\eta_{p}^{2}=0.02$, 95% CI [0.00, 0.07], with higher belief in the balanced condition. However, this was qualified by a significant pre/post by myth proportion interaction, *F*_1, 246_ = 7.55, *p* = 0.006, $\eta_{p}^{2}=0.03$, 95% CI [0.00, 0.08]. Follow-up paired *t*-tests revealed that participants in both the balanced, *t*_251_ = 19.90, *p* < 0.001, *d* = 1.25, 95% CI [1.07, 1.43], and mostly myths condition, *t*_243_ = 25.40, *p* < 0.001, *d* = 1.63, 95% CI [1.42, 1.87], significantly reduced their belief following the fact-checks; however, the reduction was larger in the mostly myths condition. There was no main effect of party congruence, and it was not involved in any interactions. There was also no main effect of Brexit congruence, and it was not involved in any interactions.

Experiment 2 replicated significant main effects of pre/post (*p* < 0.001), myth proportion (*p* = 0.010), and pre/post by myth proportion interaction (*p* = 0.014; see electronic supplementary material). There was additionally a main effect of party congruence, *F*_1, 218_ = 8.14, *p* = 0.005, $\eta_{p}^{2}=0.04$, 95% CI [0.00, 0.10], with greater myth belief when politicians and participants were aligned, and a significant pre/post by party congruence interaction, *F*_1, 218_ = 4.68, *p* = 0.032, $\eta_{p}^{2}=0.02$, 95% CI [0.00, 0.07], indicating that belief reduction was larger for party-congruent politicians due to the higher pre-correction baseline. Finally, there was also a significant main effect of Brexit congruence, *F*_1, 220_ = 20.50, *p* < 0.001, $\eta_{p}^{2}=0.09$, 95% CI [0.03, 0.16], indicating greater belief in myths from Brexit-congruent politicians. This was qualified by a significant pre/post by Brexit congruence interaction, *F*_1, 218_ = 7.26, *p* = 0.008, $\eta_{p}^{2}=0.03$, 95% CI [0.00, 0.09]. Follow-up paired *t*-tests revealed that participants reduced their belief in myth statements from both Brexit-congruent, *t*_221_ = 18.70, *p* < 0.001, *d* = 1.25, 95% CI [1.06, 1.52], and Brexit-incongruent politicians, *t*_221_ = 17.60, *p* < 0.001, *d* = 1.18, 95% CI [1.00, 1.38]; however, the reduction was larger for Brexit-congruent politicians due to the higher pre-correction baseline.

The results from both experiments clearly demonstrate that participants increased their belief in fact statements and decreased their belief in myth statements in response to the fact-checks. Additionally, across both experiments the reduction in myth beliefs was larger when participants were presented with mostly myth statements than when shown an equal number of fact and myth statements.

#### 3.1.3. Proportion of inaccurate claims

**Figure 3.**
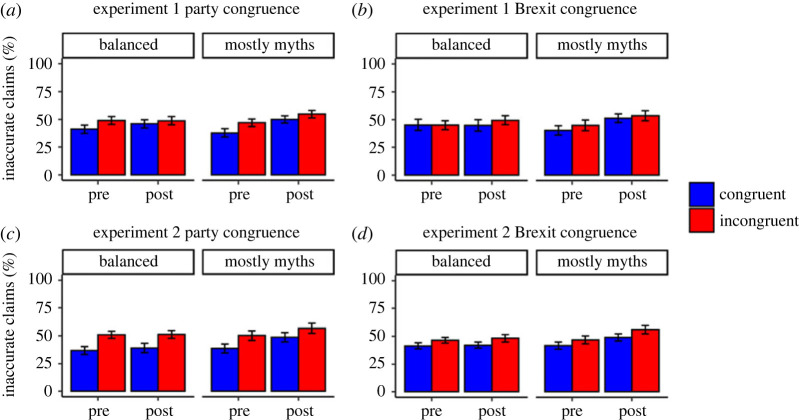
Ratings of Inaccurate Claims Politicians Make (Mean %) for Experiments 1 and 2. *Note*. Error bars represent 95% confidence intervals. Results show that after the presentation of the statements and fact-checks, participants in the mostly myths condition thought a greater proportion of politician statements were inaccurate. There is also an effect of party congruence, participants thinking politicians from the opposing political party make more inaccurate statements. A similar effect of Brexit-position congruence was significant only in Experiment 2. See text for additional details.

After analysing changes in statement beliefs, we examined how presenting the statements and fact-checks influenced perceptions of the overall veracity of the politicians, as well as whether this was impacted by political congruence and the proportion of myth statements. Participant's ratings of the proportion of claims made by the politicians that are inaccurate, for both experiments, are presented in figure 3 (also see electronic supplementary material, tables S6 and S7 for descriptive statistics).

In Experiment 1, there were significant main effects of pre/post, *F*_1, 246_ = 22.53, *p* < 0.001, $\eta_{p}^{2}=0.08$, 95% CI [0.03, 0.16] and party congruence, *F*_1, 246_ = 17.85, *p* < 0.001, $\eta_{p}^{2}=0.07$, 95% CI [0.02, 0.14], indicating greater ratings post fact-check and in case of party incongruence. There was also a significant pre/post by myth proportion interaction, *F*_1, 246_ = 8.86, *p* = 0.003, $\eta_{p}^{2}=0.03$, 95% CI [0.00, 0.09]. Follow-up paired *t*-tests revealed that in the mostly myths condition, participants increased their estimated number of inaccurate claims following presentation of the statements and fact-checks, *t*_243_ = 6.50, *p* < 0.001, *d* = 0.42, 95% CI [0.29, 0.55], whereas in the balanced condition there was no significant change, *t*_251_ = 1.29, *p* = 0.197, *d* = 0.08, 95% CI [−0.04, 0.21]. Pre/post also interacted with party congruence, *F*_1, 246_ = 5.28, *p* = 0.022, $\eta_{p}^{2}=0.02$, 95% CI [0.00, 0.07]. Follow-up paired *t*-tests revealed that participants increased their ratings for both party-congruent, *t*_247_ = 4.93, *p* < 0.001, *d* = 0.31, 95% CI [0.19, 0.46], and party-incongruent politicians, *t*_247_ = 2.27, *p* = 0.024, *d* = 0.14, 95% CI [0.02, 0.28], after the statements and fact-checks; however, this effect was larger for party-congruent politicians, due to a lower pre-intervention baseline. There was no significant main effect of Brexit congruence, and it was not involved in any interactions.

Experiment 2 replicated the significant main effects of pre/post (*p* = 0.002), party congruence (*p* < 0.001), and the pre/post by myth proportion interaction (*p* = 0.019) with significant increases again occurring only in the mostly myths condition (see electronic supplementary material). Unlike Experiment 1, in Experiment 2 there was also a significant main effect of Brexit congruence, *F*_1, 218_ = 35.96, *p* < 0.001, $\eta_{p}^{2}=0.14$, 95% CI [0.07, 0.23], with higher estimates in the incongruent condition. There were no other significant interactions or main effects.

In sum, following the fact-checks, participants thought a greater proportion of the politician statements were inaccurate but only if they were shown mostly myth statements. Additionally, participants thought politicians from the opposing political party made more inaccurate statements (in both experiments) and in Experiment 2 this was also found for politicians with the opposing Brexit position.

### 3.2. Attitudes towards politicians

To answer our research questions about how fact-checks influence attitudes towards politicians more broadly, we examined how voting intentions and feelings towards politicians changed in response to the presentation of the statements and fact-checks. We also examined the effects of political congruence and the proportion of myth statements. Before and after being presented with the statements and fact-checks, participants rated their voting intentions and feelings for all politicians. In the interests of brevity and clarity, we report and focus on the results for politicians who presented statements.^2^ Additionally, because there were no significant effects of myth proportion and it did not meaningfully interact with any other factors, it is not included as a factor in the figures.

#### 3.2.1. Voting intentions

**Figure 4.**
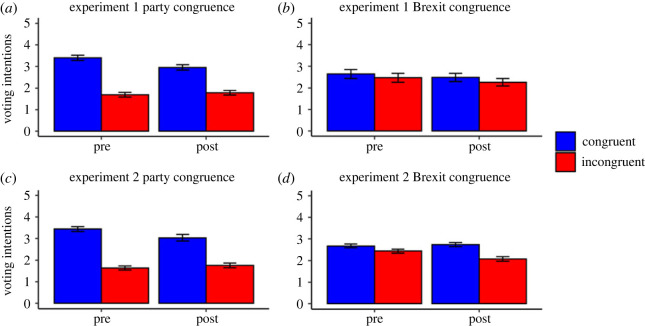
Voting Intentions (Means) for Experiments 1 and 2. *Note.* Error bars represent 95% confidence intervals. Results show that participants decreased their voting intentions following the statements and fact-checks, but only for politicians from the party they support. Additionally, the impact of party congruence was greater than the impact of Brexit congruence. See text for additional details.

Voting intentions for both experiments are presented in figure 4 (also see electronic supplementary material, tables S8 and S9 for descriptive statistics).

In Experiment 1, there were significant main effects of pre/post, *F*_1, 246_ = 16.44, *p* < 0.001, $\eta_{p}^{2}=0.06$, 95% CI [0.02, 0.13] and party congruence, *F*_1, 246_ = 304.03, *p* < 0.001, $\eta_{p}^{2}=0.55$, 95% CI [0.48, 0.62]; these indicated lower voting intentions after presentation of the statements and fact-checks and for politicians of the non-supported party. These main effects were qualified by a significant pre/post by party congruence interaction, *F*_1, 246_ = 42.66, *p* < 0.001, $\eta_{p}^{2}=0.15$, 95% CI [0.08, 0.23]. Follow-up paired *t*-tests revealed that participants significantly reduced their voting intentions for party-congruent politicians following the statements and fact-checks, *t*_247_ = 6.47, *p* < 0.001, *d* = 0.41, 95% CI [0.29, 0.53], whereas for party-incongruent politicians participants seemed to increase their voting intentions following the statements and fact-checks, although this change failed to reach significance, *t*_247_ = 1.83, *p* = 0.068, *d* = 0.12, 95% CI [0.00, 0.23]. There was also a significant main effect of Brexit congruence, *F*_1, 244_ = 6.96, *p* = 0.009, $\eta_{p}^{2}=0.03$, 95% CI [0.00, 0.08], with lower voting intentions for politicians of the opposing Brexit position. There were no other significant main effects or interactions.

Experiment 2 replicated the significant main effects of pre/post (*p* < 0.001), party congruence (*p* < 0.001), and the pre/post by party congruence interaction (*p* < 0.001; see electronic supplementary material). This interaction again occurred because participants decreased their voting intentions for party-congruent politicians (*p* < 0.001), but slightly increased their voting intentions for party-incongruent politicians (*p* = 0.017). The main effect of Brexit congruence was also replicated (*p* < 0.001). Unlike Experiment 1, this main effect was qualified by a significant pre/post by Brexit congruence interaction, *F*_1, 218_ = 29.74, *p* < 0.001, $\eta_{p}^{2}=0.12$, 95% CI [0.05, 0.20]. Follow-up paired *t*-tests revealed that participants decreased their voting intentions for Brexit-incongruent politicians following presentation of the statements and fact-checks, *t*_221_ = 5.58, *p* < 0.001, *d* = 0.37, 95% CI [0.26, 0.48], whereas there was no significant change for Brexit-congruent politicians, *t*_221_ = 1.10, *p* = 0.272, *d* = 0.07, 95% CI [−0.07, 0.21]. There were no other significant main effects or interactions.

The findings for voting intentions show that participants decreased their voting intentions following the presentation of the statements and fact-checks, but only for politicians from the party they support. Additionally, in both experiments the effect of party congruence was larger than the effect of Brexit congruence (as evidenced by the non-overlapping 95% CIs for the effect sizes).

#### 3.2.2. Feelings

**Figure 5.**
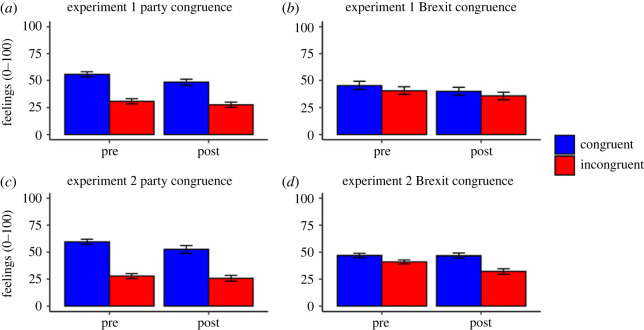
Feelings Towards Politicians (Means) for Experiments 1 and 2. *Note.* Error bars represent 95% confidence intervals. Results show that following the statements and fact-checks participants felt more negatively towards politicians from the party they support. Additionally, the impact of party congruence was greater than the impact of Brexit congruence. See text for additional details.

Feelings towards politicians for both experiments are presented in figure 5 (also see electronic supplementary material, tables S10 and S11 for descriptive statistics).

In Experiment 1, there were significant main effects of pre/post, *F*_1, 246_ = 29.65, *p* < 0.001, $\eta_{p}^{2}=0.11$, 95% CI [0.05, 0.18], and party congruence, *F*_1, 246_ = 160.25, *p* < 0.001, $\eta_{p}^{2}=0.39$, 95% CI [0.31, 0.47]; these indicated cooler feelings after presentation of the statements and fact-checks, and towards politicians of the non-supported party. These main effects were qualified by a significant pre/post by party congruence interaction, *F*_1, 246_ = 5.23, *p* = 0.023, $\eta_{p}^{2}=0.02$, 95% CI [0.00, 0.07]. Follow-up paired *t*-tests revealed that although participants had cooler feelings towards both party-congruent, *t*_247_ = 5.25, *p* < 0.001, *d* = 0.33, 95% CI [0.23, 0.45], and party-incongruent politicians after the statements and fact-checks, *t*_247_ = 2.60, *p* = 0.010, *d* = 0.17, 95% CI [0.04, 0.30], this effect was larger for party-congruent politicians.

There was also a significant main effect of Brexit congruence, *F*_1, 244_ = 5.99, *p* = 0.015, $\eta_{p}^{2}=0.02$, 95% CI [0.00, 0.07], indicating warmer feelings towards politicians of the same Brexit position. This was qualified by a marginal three-way interaction between pre/post, Brexit congruence and myth proportion, *F*_1, 244_ = 4.20, *p* = 0.041, $\eta_{p}^{2}=0.02$, 95% CI [0.00, 0.06]. Follow-up ANOVAs, split by myth proportion, found a main effect of Brexit congruence in the mostly myths, *F*_1,120_ = 5.62, *p* = 0.019, $\eta_{p}^{2}=0.04$, 95% CI [0.00, 0.14], but not the balanced condition, *F*_1,124_ = 1.38, *p* = 0.243, $\eta_{p}^{2}=0.01$, 95% CI [0.00, 0.07]. There were significant main effects of pre/post in both conditions (*p*s < 0.001) but there were no significant interactions between pre/post and Brexit congruence in either myth proportion condition.

Experiment 2 replicated the significant main effects of pre/post (*p* < 0.001), party congruence (*p* < 0.001), and the pre/post by party congruence interaction (*p* = 0.010). Participants' feelings towards party-congruent politicians diminished after the statements and fact-checks, *t*_225_ = 5.22, *p* < 0.001, *d* = 0.35, 95% CI [0.22, 0.48], but unlike Experiment 1, there was no significant change for party-incongruent politicians, *t*_217_ = 1.60, *p* = 0.110, *d* = 0.11, 95% CI [−0.03, 0.25]. Experiment 2 also replicated the main effect of Brexit congruence (*p* < 0.001). This was qualified by a pre/post by Brexit congruence interaction, *F*_1, 218_ = 28.67, *p* < 0.001, $\eta_{p}^{2}=0.12$, 95% CI [0.05, 0.20]. Participants had cooler feelings towards Brexit-incongruent politicians after the statements and fact-checks, *t*_221_ = 6.47, *p* < 0.001, *d* = 0.43, 95% CI [0.31, 0.56], whereas there was no significant difference for Brexit-congruent politicians, *t*_221_ = 0.16, *p* = 0.876, *d* = 0.01, 95% CI [−0.12, 0.14]. There were no other significant main effects or interactions.

The results for feelings towards politicians indicate that participants felt more negatively towards politicians following the presentation of the statements and fact checks, particularly for politicians from the party they support. As with voting intentions, the effect of party congruence was larger than the effect of Brexit congruence (non-overlapping 95% CIs).

## 4. Discussion

This study aimed to investigate how UK participants update their belief in political statements in response to fact-checks as well as whether the fact-checks would have flow-on effects for participants’ attitudes towards the politicians. Additionally, we examined whether the proportion of inaccurate claims presented had an impact on belief and attitude changes in response to the fact-checks. Finally, we investigated how divisions based on party affiliation and Brexit position impacted both overall beliefs and political attitudes, and changes in beliefs and attitudes in response to the fact-checks.

### 4.1. Belief change

Overall, the key finding for belief statements is that participants were able to clearly distinguish between facts and myths following the fact-checks. Even though participants tended to believe more in statements from politically aligned politicians (particularly prior to the fact-checks), after the fact-checks participants greatly reduced their myth beliefs and greatly increased their fact beliefs (despite similar belief levels for the two item types prior to the fact-checks). This result is consistent with the prior literature on political fact-checking, which has demonstrated that people update their beliefs about the accuracy of statements in response to fact-checks, even for false (true) statements from an aligned (misaligned) politician [6–8,17]. This same pattern was found regardless of whether the political alignment was based on party affiliation or Brexit position, providing further support for the notion that the effects of motivated reasoning are not so strong that they overwhelm voters' willingness or ability to update their beliefs when presented with affirming or correcting information.

There was also an effect of myth proportion on myth belief ratings. In both experiments, participants in the mostly myths condition reduced their myth beliefs to a greater extent than those in the balanced condition. This may represent a general shift in perceptions of the accuracy of the statements made by the politicians. Because participants in the mostly myths condition were shown four inaccurate statements that were corrected and only one accurate statement that was affirmed, they may have viewed the associated politicians as generally less trustworthy and therefore reduced their belief for the myth statements to a greater extent.

### 4.2. Politician inaccuracy ratings

Findings for the overall judgements of the truthfulness of politicians again found effects of divisions and the proportion of myths presented. In both experiments, participants indicated that politicians from the opposing political party made a greater proportion of inaccurate statements. There was no effect of Brexit alignment on overall truthfulness ratings in Experiment 1. However, in Experiment 2, which placed greater emphasis on the politicians’ Brexit positions, participants also indicated that politicians with the opposing Brexit position make more inaccurate statements. Additionally, in both experiments, participants in the mostly myths condition said that politicians make more inaccurate claims after the statements and fact-checks were presented, whereas in the balanced condition there was no significant change. This finding is consistent with the proposed explanation for why myth belief ratings were lower in the mostly myths condition, that is, that the mostly myths condition led participants to view the politicians as less trustworthy in general.

### 4.3. Political attitudes

Participants had greater voting intentions and more positive feelings for politicians who were aligned with the political party and Brexit position that they support. Of the two, party congruence was consistently the more impactful, as evidenced by the consistently larger effect sizes than for Brexit congruence (non-overlapping 95% CIs). Given the focus on the level of Brexit related division that has occurred following the EU referendum, these results may seem somewhat surprising [26,27]. However, much of this focus is likely due to Brexit emerging as a new dividing line and therefore garnering greater interest. Indeed, even within research and surveys that highlight the Brexit divide, the data still clearly show that division along party lines is as strong or stronger than division based on Brexit position [9,23,24]. Partisan divides may be stronger because they are generally longer-standing and also encompass views on a wide variety of political topics such as jobs, education, income inequality, and the way the political system works [24].

As in Swire-Thompson *et al*. [8], we found that decreases in voting intentions and feelings were only significant for party-congruent politicians, likely because low baseline voting intentions and feelings towards party-incongruent politicians meant there was limited room for decreases. This differs from the findings of Aird *et al*. [6], who found a reduction for both party-congruent and party-incongruent politicians. The floor effects for party-incongruent politicians may reflect greater levels of partisanship in the USA and the UK than in Australia. However, the effect sizes for the changes for party-congruent politicians were similar to Aird *et al*., and much larger than Swire-Thompson *et al*. found in the USA. This may suggest that, like Australia, UK participants care about the honesty of their politicians and are more willing to change their political attitudes. However, in Experiment 2, the opposite pattern was consistently found for Brexit congruence, with voting intentions and feelings towards Brexit-incongruent politicians decreasing following the statements and fact-checks. This finding is consistent with the idea that politicians' party affiliations were generally better known and more salient than their Brexit positions. The politicians’ biographies, which mentioned their party affiliation and highlighted their Brexit positions, were shown before the fact-checks and statements, but after participants provided their initial voting intentions and feelings. Therefore, the post fact-check reductions for Brexit-incongruent politicians may have occurred as a result of participants learning or confirming the Brexit position of the politicians.

Another key research question was how the presentation of the statements and fact-checks impacted attitudes towards politicians, as well as whether the proportion of accurate to inaccurate statements (balanced versus mostly myths) moderated this effect. However, unlike previous studies [6,8], the proportion of inaccurate statements did not affect voting intentions and feelings. Instead, we found that, regardless of condition, the presentation of the statements and fact-checks tended to decrease voting intentions and led to more negative feelings towards the politicians.

### 4.4. Limitations and future directions

A potential limitation, and alternative explanation, for the political attitude findings in our study is that the party affiliation of the politicians may have been better known and more salient to participants than their Brexit positions. Unlike previous studies [6–8], we did not focus on party leaders and therefore participants may have known less about the politicians included. Additionally, the impact of presenting statements and fact-checks may differ depending on the profile of the politician and pre-existing attitudes towards them (see electronic supplementary material for results for each individual politician). However, the politicians included had relatively high and well-matched Google search traffic and played high-profile roles in the Brexit debate. Therefore, if Brexit position was highly salient and impactful, then it should have impacted attitudes and judgements within the study. Nonetheless, knowing a politician's Brexit position may require greater political knowledge than knowing their party affiliation. This could be examined in future research by testing whether participants can successfully indicate the party affiliation and Brexit position of politicians. Additionally, in both experiments the politicians' party affiliations were mentioned in their biography, whereas their Brexit positions were only highlighted in Experiment 2. Differences between the results of Experiments 1 and 2 provide some support for this explanation, with a larger effect of Brexit alignment in Experiment 2 (non-overlapping 95% CIs). However, it is important to note that even in Experiment 2, party congruence still had a larger effect on voting intentions and feelings. Another potential issue is the timing of data collection for the current study. UK political parties underwent considerable change in response to the Brexit referendum, which may have brought party affiliation and Brexit position into closer alignment and thereby reduced the impact of Brexit as a cross-cutting issue [28]. Additionally, data for the present study were collected after the 2019 General Election and the UK's exit from the EU; as such, participants may have seen the Brexit issue as somewhat resolved [29].

It is also important to consider the extent to which the sample used within the study is representative of the broader UK population. Data were collected via Prolific and were not representative in terms of political views (more Labour Party and Remain supporters) and education (more highly educated). However, previous research has found that results from online convenience samples consistently replicated in nationally representative samples [30–33], and our sample is generally comparable to the samples used in previous research conducted in the USA [7,8] and Australia [6]. Nonetheless, to allow for proper cross-cultural comparisons of responses to political misinformation, it would be beneficial for future work to collect large-scale representative samples from multiple countries.

Another exciting avenue for future work is to examine the impact of issue-based political divisions across a wider range of countries and issues. Recent years have seen the emergence of several issue-based divisions that do not map neatly onto existing partisan divides. For example, 29% of US Republicans opposed the US Supreme Court decision to overturn Roe v. Wade and 38% said abortion should be legal in most or all cases [34]. Relatedly, despite being a conservative state, Kansas voted strongly against an abortion ban earlier this year [35]. Similarly, the recent Australian election saw the conservative-leaning Liberal Party lose several of its heartland seats to independents who campaigned on the issue of climate change [36]. Therefore, it would be useful for future work to examine the impact of these issue-based political divisions on how people view politicians and to compare these issue-based divisions with divisions based on party affiliation.

It would also be beneficial for future research to examine the extent to which findings from the current study, and similar studies on political fact-checking, generalize to political statements more broadly. Consistent with previous research [6–8], we presented either equal numbers of accurate and inaccurate statements or disproportionately more inaccurate statements. However, it is unclear how participants would respond if they were presented with a disproportionate number of accurate statements from politicians. For example, politicians who presented mostly true, or only true statements, may receive a benefit in terms of participants' attitudes and/or voting intentions. It would be beneficial for future work to examine responses to a broader range of ratios of accurate and inaccurate statements, as well as whether impacts differ depending on baseline expectations about how often politicians make inaccurate claims (e.g. do attitudes decrease (increase) depending on whether the proportion of inaccurate statements is higher (lower) than expected).

Similarly, the types of political statements that are amenable to fact-checking tend to refer to a narrow or specific claim (e.g. the false claim that immigration to the UK led to increased unemployment). People may be willing to change beliefs about these specific statements because they can do so while still maintaining their core beliefs and attitudes. By contrast, by their very nature, broader statements (e.g. immigration is causing the UK problems) that directly challenge participants’ core political beliefs or attitudes also tend to be much less amenable to fact-checking because of the lack of specificity (e.g. which problems are being caused, which kind of immigration, are benefits also being considered, etc.). This is a potential issue because these broad statements may be more prone to motivated reasoning and resistance to change than narrower claims [13–15,37,38]. For example, Ecker & Ang [39] found that participants were more resistant to updating a belief that related to a general statement (politically aligned politicians are more prone to misconduct) than a belief related to a specific incident (a single instance of misconduct by a politically aligned politician). Additionally, fact checking is primarily conducted by named media organizations. Given public perceptions of political bias in the media [40,41], real-world fact checks may be less effective when they come from a named media source. Thus, political worldview may present a more significant barrier to belief updating in the real world.

## 5. Conclusion

Overall, the results of this study clearly show that fact-checks can be effectively used to counter political misinformation, adding to the existing evidence base and showing that these results generalize to a UK context [6–8,17]. We also found that, if politicians disproportionately present misinformation, participants reduce their belief in inaccurate statements to a greater extent. These views at least partially extend to estimates of general politician accuracy, with disproportionate amounts of misinformation also leading to more negative judgements about the day-to-day accuracy of the politicians. Furthermore, feelings and voting intentions for party-congruent politicians were lower following the statements and fact-checks even if equal numbers of accurate and inaccurate statements were presented. This finding suggests that when politicians make statements, voters expect them to do better than providing accurate information half the time. Finally, although we found divisions based on both party and Brexit alignment, these effects were much stronger for party alignment, highlighting that even though new divisions have emerged in UK politics, the old divides remain entrenched and dominant.

## Ethics

All procedures were approved by the Faculty of Science Ethics Committee at the University of Bristol; Ethics ID: 98264. All participants provided informed consent prior to participating.

## Data Availability

Data, analysis code and materials for this study are available on the Open Science Framework: https://osf.io/tnzsa/. The data are provided in electronic supplementary material [42].

## Footnotes

1 The main findings and conclusions of the study are robust to these exclusions. However, the significance of some effects changes. See electronic supplementary material, table S1 for full details.

2 See electronic supplementary material for the results for party leaders and the politicians not associated with the presented statements.
